# Expression and clinical significance of PD-L1, B7-H3, B7-H4 and TILs in human small cell lung Cancer (SCLC)

**DOI:** 10.1186/s40425-019-0540-1

**Published:** 2019-03-08

**Authors:** Daniel Carvajal-Hausdorf, Mehmet Altan, Vamsidhar Velcheti, Scott N. Gettinger, Roy S. Herbst, David L. Rimm, Kurt A. Schalper

**Affiliations:** 1 Department of Pathology, Yale School of Medicine, New Haven, USA; 2 Anatomia Patologica, Clinica Alemana, Facultad de Medicina Universidad del Desarrollo, Santiago, Chile; 3 Medical Oncology, Yale School of Medicine and Yale Cancer Center, 333 Cedar St. FMP117, New Haven, CT 06520-8023 USA; 4 Thoracic Oncology, MD Anderson Cancer Center, Camden, USA; 5 Thoracic Oncology, New York University, New York, USA

## Abstract

**Background:**

Small cell lung cancer (SCLC) accounts for 10–15% of all lung malignancies and its prognosis is dismal. Although early studies have shown promising clinical activity of immune checkpoint blockers, the immune composition and expression of potentially actionable immunostimulatory targets in this malignancy are poorly understood.

**Methods:**

Using multiplexed quantitative immunofluorescence (QIF), we measured the levels of 3 different B7 family ligands PD-L1, B7-H3, B7-H4 and major tumor infiltrating lymphocyte (TIL) subsets in 90 SCLC samples represented in tissue microarray format. Associations between the marker levels, clinicopathological variables and survival were studied.

**Results:**

PD-L1 protein was detected in 7.3%, B7-H3 in 64.9% and B7-H4 in 2.6% of SCLC cases. The markers showed limited co-expression and were not associated with the level of TILs, age, gender and stage. Elevated B7-H4 was associated with shorter 5-year overall survival. The levels of CD3+, CD8+ and CD20+ TILs and the ratio of total/effector T-cells were significantly lower in SCLC than in non-small cell lung cancer. High levels of CD3+, but not CD8+ or CD20+ TILs were significantly associated with longer survival.

**Conclusions:**

Taken together, our study indicate variable expression and clinical role of B7-family ligands in SCLC with predominant expression of the candidate target B7-H3 and the presence of a limited cytotoxic anti-tumor immune response. These results support the evaluation of B7-H3 blockers and/or pro-inflammatory therapies in SCLC.

## Background

Small cell lung cancer (SCLC) accounts for approximately 10–15% of all lung carcinomas and comprise high-grade neuroendocrine tumors with aggressive clinical course and prominent association with tobacco use [1–3]. To date, there are limited therapeutic options and the prognosis is ominous with 5-year survival rates of only around 3–6% for extensive stage SCLC [1]. Molecular characterization of SCLC has revealed an extremely high nonsynonymous mutational rate and the presence of deleterious variants in the tumor suppressor genes TP53 and RB1 virtually in all cases [3, 4]. In addition, SCLCs contain relatively low frequency of mutations in actionable oncogenes, limiting the therapeutic options [5]**.**

Immunostimulatory therapies blocking the PD-1 axis produce prominent and lasting clinical responses in nearly 20% of non-small cell carcinomas (NSCLC), the most common form of lung cancer [6–9]. The clinical benefit to PD-1 axis blockers is associated with tumor PD-L1 expression, pre-existing anti-tumor immune response and increased tumor mutational burden [6, 8–11]. Although preliminary data from ongoing trials using antagonistic PD-1 and CTLA-4 antibodies in heavily pre-treated SCLCs suggests limited activity of monotherapy regimens, combination PD-1/CTLA-4 immune checkpoint blockade show encouraging results with objective responses in up to ~ 30% of cases [12, 13]. Despite these results, little is known about the immune composition of SCLC and most studies characterizing immune cells or targets have used qualitative/subjective methods. Identification of dominant immune cell populations and/or expression of candidate immunotherapy targets in this tumor could support optimal design and interpretation of clinical trials.

PD-L1 protein expression has been found in a highly variable proportion of SCLC ranging from 0% in one study including 61 samples [14] to 71.6% in another study with 102 cases [15]. The biological determinants for this discrepancy remain unknown but are likely due to technical differences or limitations of the methods used. Here, we used validated assays and multiplexed quantitative immunofluorescence (QIF) to objectively measure and assess the clinical impact of PD-L1, B7-H3, B7-H4 and major TIL subpopulations in human SCLCs.

## Methods

### Patients, cohorts and tissue microarrays

Samples from a retrospectively collected SCLC cohort from Yale University represented in 2 tissue microarrays (TMAs) (YTMA57 and YTMA259) totaling 90 cases were used. Detailed clinico-pathological characteristics of the cohorts were collected form surgical pathology reports and clinical records. TMAs were prepared using 0.6 mm tissue cores, each in 2-fold redundancy using standard procedures [16, 17]. The actual number of samples analyzed for each marker is lower than the total samples in the cohort due to unavoidable loss of tissue, absence or limited tumor cells in some spots as is commonly seen in TMA studies or incomplete clinicopathologic annotation. All tissue was used after approval from the Yale Human Investigation Committee protocols #9505008219 and #1608018220, which approved the patient consent forms or in some cases waiver of consent.

### Multiplexed quantitative immunofluorescence (QIF)

We measured the levels of PD-L1 (E1L3N, Cell Signaling technology), B7-H3 (D9M2L, Cell Signaling Technology), B7-H4 (D1M8I, Cell Signaling Technology), CD3 (clone E272, Novus Biologicals), CD8 (clone C8/144B, DAKO), CD20 (clone L26, DAKO) and pancytokeratin (AE1/AE3, DAKO) using QIF in TMA slides containing the cohort cases. PD-L1, B7-H3 and B7-H4 were stained in serial sections from the TMA blocks using a previously described protocol with simultaneous detection of cytokeratin and 4′,6-diamidino-2-phenylindole (DAPI) [18, 19]. Briefly, antigen retrieval was with citrate buffer pH 6.0 for 20 min at 97 °C in a pressure-boiling container and blocking was performed with 0.3% bovine serum albumin in 0.05% Tween solution for 30 min. Primary antibodies were incubated overnight using a dilution of 1:1600 for PD-L1, 1:500 for B7-H3 and 1:200 for B7-H4. Stringent validation and optimization of these assays using cell line transfectants and endogenous human tissue controls has been reported by our group [18, 20, 21]. Secondary antibody for cytokeratin was Alexa 546-conjugated goat anti-mouse or anti-rabbit (Invitrogen Molecular Probes, Eugene, OR, USA). Cyanine 5 (Cy5) directly conjugated to tyramide (FP1117; Perkin-Elmer) at a 1:50 dilution was used for target antibody detection.

CD3, CD8, CD20 and cytokeratin were simultaneously stained using a sequential staining protocol, as previously described [16, 20, 22]. Briefly, TMA sections were deparaffinized and subjected to antigen retrieval using pH = 8.0 EDTA buffer (Sigma-Aldrich, St Louis, MO, USA) and boiled for 20 min at 97 °C in a pressure-boiling container (PT module, Lab Vision, Thermo Scientific, Waltham, MA, USA). Slides were then incubated with dual endogenous peroxidase block (DAKO #S2003, Carpinteria, CA, USA) for 10 min at room temperature and subsequently with a blocking solution containing 0.3% bovine serum albumin in 0.05% Tween solution for 30 min. Residual horseradish peroxidase activity between incubations with secondary antibodies was eliminated by exposing the slides twice for 7 min to a solution containing benzoic hydrazide (100 mM) and hydrogen peroxide (50 mM) in PBS. Isotype specific, fluorophore-conjugated secondary antibodies were used for signal detection and nuclei were highlighted using DAPI.

### Fluorescence signal quantification and cases stratification

Quantitative measurement of the fluorescent signal was performed using the AQUA® method of QIF, as previously reported [18, 20, 23]. Briefly, the QIF score of each fluorescence channel was calculated by dividing the target marker pixel intensities by the area of the desired compartment. Scores were normalized to the exposure time and bit depth at which the images were captured, allowing scores collected at different exposure times to be comparable. The immune target scores and TIL markers considered the signal detected in the whole tissue compartment using an adjusted DAPI mask. Cases were considered as target expressers when the QIF score was above the signal detection threshold determined using the negative control preparations and visual inspection. For stratification, the marker levels were classified as high/low using the top 25-percentile of the cohort scores as stratification cut-point.

### Statistical analyses

QIF signal differences between groups were analyzed using t-test for continuous variables and chi-square test for categorical variables. Linear regression coefficients were calculated to determine the association between continuous scores. Survival analysis based on marker expression was performed using Kaplan-Meier analyses with log rank test and overall survival as endpoint. Statistical significance was considered at *P* < 0.05 and analyses were performed using JMP® Pro software (version 9.0.0, 2010, SAS Institute Inc.) and GraphPad Prism v6.0 for Windows (GraphPad Software, Inc). All statistical tests were two-sided.

## Results

### Expression of PD-L1, B7-H3, B7-H4 and TILs in SCLC

**Fig. 1:**
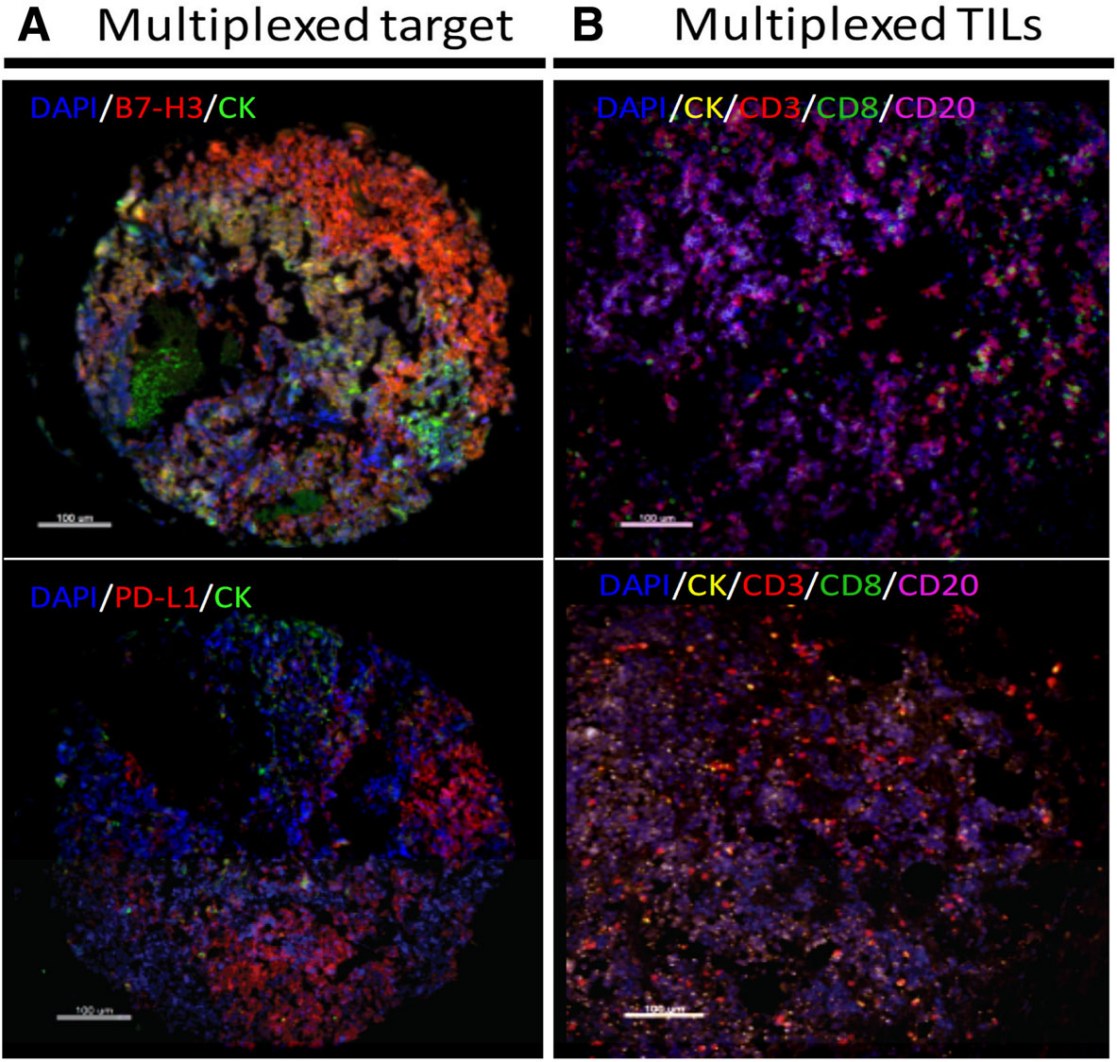
Detection of immune targets and TILs in SCLC using multiplex quantitative fluorescence. **a** Representative fluorescence pictures showing B7-H3 (upper panel) and PD-L1 (lower panel) protein expression in SCLC. The target signal (red fluorescence) is predominantly located in tumor cells. **b** Representative fluorescence pictures showing the signal for DAPI (blue), cytokeratin (CK, green), CD3 (red), CD8 (green) and CD20 (magenta) staining in SCLC. Bar = 100 um

We previously validated and optimized assays for detection of PD-L1, B7-H3, B7-H4 and TIL markers using formalin-fixed, paraffin-embedded (FFPE) preparations from human tissue samples and cell line transfectants [17, 20–23]. As expected for SCLC, positive staining for cytokeratin was focal and frequently showed a perinuclear dot-like staining pattern (Fig. 1). PD-L1 and B7-H3 were predominantly recognized in tumor cells with cytoplasmic and membranous staining (Fig. 1a). Prominent B7-H4 positivity was infrequently recognized and showed relatively low signal with a focal staining pattern. Expression of TIL markers showed predominance of CD3+ T-cell staining with CD8+ and CD20+ cells displaying low levels in the cohort. Representative examples from cases with prominent CD3+ TILs or CD20+ B-cell infiltrates are shown in Fig. 1b**.**

**Fig. 2:**
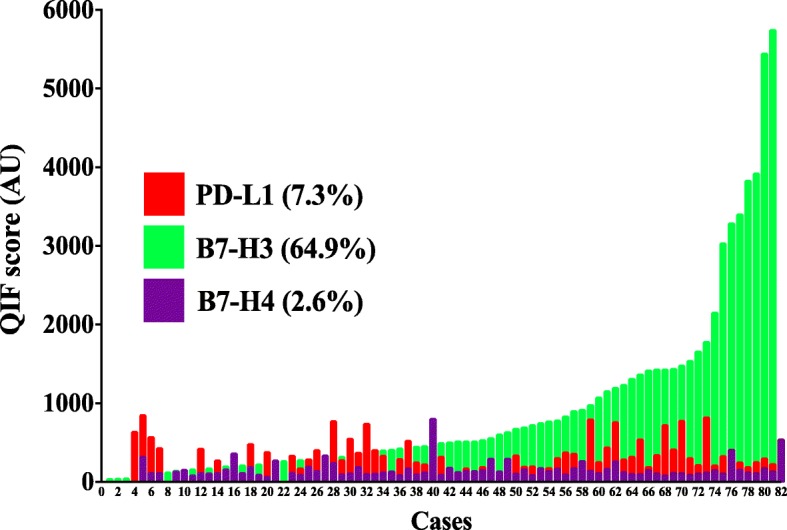
Levels of different immune targets in SCLC. Distribution of PD-L1 (red), B7-H3 (green) and B7-H4 (magenta) QIF scores in SCLCs from Yale. The frequency of expression for each marker is indicated in parenthesis. The cut-point used to define expression was the signal detection threshold. AU = Arbitrary units of fluorescence

**Fig. 3:**
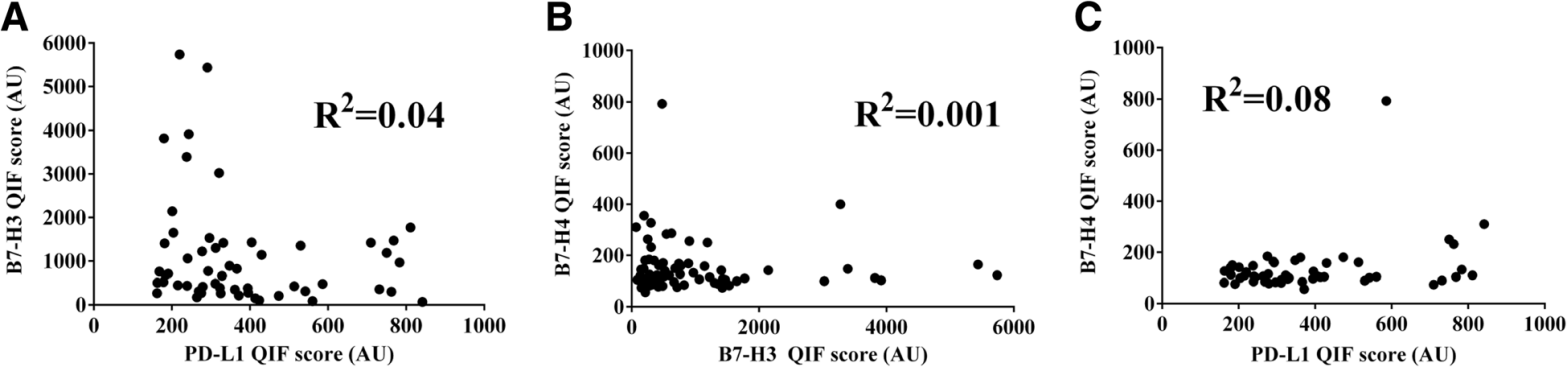
PD-L1, B7-H3 and B7-H4 are infrequently co-expressed in SCLC. A-C) Histograms showing the levels of PD-L1, B7-H3 and B7-H4 protein in small cell lung carcinomas from the Yale cohort. The linear regression coefficients (R^2^) of the scores between each marker pair are indicated within the charts

Using the visual detection threshold by pathologists-based analysis, we detected tumor-cell PD-L1, B7-H3 and B7-H4 in 7.3, 64.9 and 2.6% of cases in the cohort (Fig. 2). In the QIF analysis, PD-L1 and B7-H4 show relatively low scores, while B7-H3 had a wider range with cases displaying prominently higher signal. Overall, the levels of B7-H3 were 2.3 fold higher than PD-L1 (mean QIF score 894 vs 386, *P* = 0.02) and 5.8 fold higher than B7-H4 (mean QIF score 894 vs 155, *P* < 0.001). Notably, the levels of the targets showed limited correlation with PD-L1 and B7-H3 showing minimal co-expression consistent with a mutually exclusive expression pattern (Fig. 3a).

**Fig. 4:**
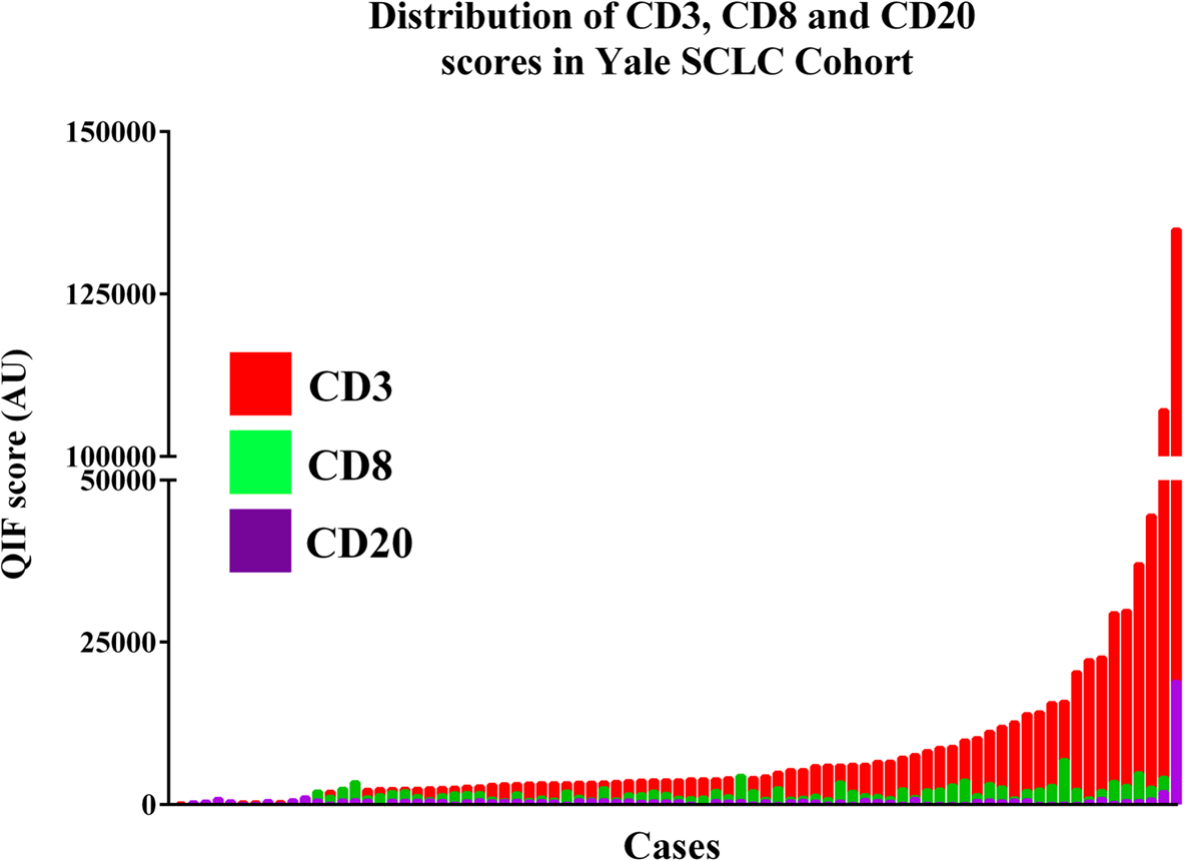
Levels of TIL subpopulations in SCLC. Distribution of CD3 (red), CD8 (green) and CD20 (magenta) QIF scores in SCLCs from the Yale cohort. Cases were stratified using the median score of each marker as stratification cut-point. AU = Arbitrary units of fluorescence

Expression of the TIL markers showed a wide range and continuous score distribution with 16% of cases displaying undetectable B and T-cell infiltration **(**Fig. 4**)**. CD3 showed the highest dynamic range of all markers and was detected in 94% of specimens. CD8+ T-cell infiltration was identified in 67% of cases and CD20+ B-lymphocyte signal was seen only in 11% of cases.

### Tumor immune infiltration of SCLC and comparison with NSCLC

**Fig. 5:**
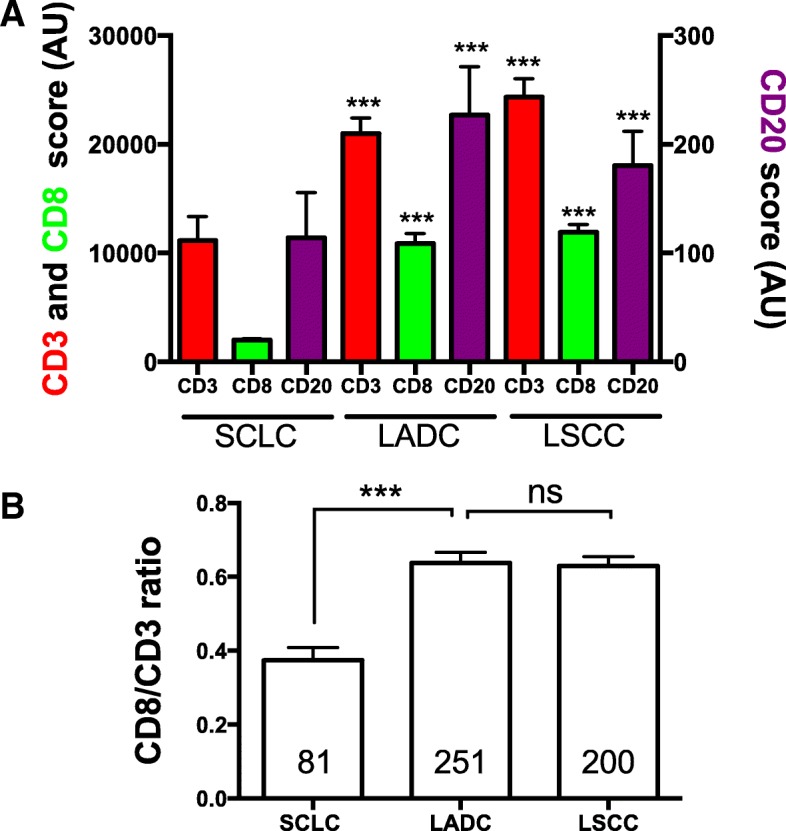
Levels of TIL subpopulations in SCLC and major NSCLC subtypes. **a** Chart showing the levels of CD3 (red), CD8 (green) and CD20 (magenta) in SCLC (left), primary lung adenocarcinomas (LADC, center) and lung squamous cell carcinomas (LSCC, right). Each bar depicts the median +/− SEM. The levels of TILs in NSCLC subtypes were obtained previously using the same multiplexing protocol [22]. **b** Chart showing the ratio of CD8/CD3 signal in SCLCs (left), LADCs (center) and LSCCs (right). The number of cases is indicated within each bar. *** = *P* < 0.001; ns = not significant. AU = Arbitrary units of fluorescence

To evaluate the TIL scores of SCLCs in the context of other lung cancer subtypes, we compared the marker levels with those obtained in retrospective cohorts of lung adenocarcinomas (LADC) and lung squamous cell carcinomas (LSCC) measured using the same assay and analysis platform [22]. As shown in Fig. 5a**,** SCLCs showed significantly lower levels of all TIL markers than LADC and LSCC (*P* = 0.01-*P* < 0.0001). The most prominent difference was in CD8 level that was 5.4 fold lower than in LADC and 6-fold lower than in LSCC. Notably, the CD3/CD8 ratio was also prominently lower in SCLC than in the major NSCLC subsets, suggesting the presence of a less cytotoxic T-cell profile in this malignancy (Fig. 5b, mean CD3/CD8 ratio of 0.37 vs 0.63 in LADC and 0.62 in LSCC, *P* < 0.001).

### Association of the markers with clinicopathologic variables and survival

**Fig. 6:**
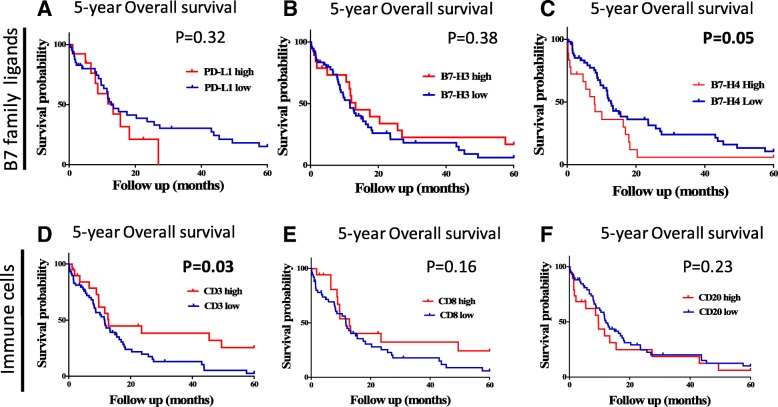
Association between the levels of B7 family ligands, TIL subsets and survival in SCLC. Kaplan-Meier graphical analysis of the 5-year overall survival in patients with SCLC from the Yale cohort. **a** Survival based on the expression of the immune ligands PD-L1 (left), B7-H3 (center) and B7-H4 (right). **b** Survival based on the expression of the TIL markers CD3 (left), CD8 (center) and CD20 (right). The respective log-rank *P* values are indicated in the chart

Elevated expression of PD-L1, B7-H3 or B7-H4 (scores within the top signal quartile) were not significantly associated with major clinicopathologic variables or TIL markers in the cohort (Table 1). As expected, the levels of CD3 were positively associated with CD8, but there was no relationship between CD3 or CD8 and CD20 in the tumors. High levels of CD20+ B-cells were more commonly seen in samples from female patients (14 of 23 [37.8%] vs 6 of 38 [13.6%], *P* = 0.01). High PD-L1 or B7-H3 protein levels were not significantly associated with 5-year overall survival (Fig. 6a-b). However, elevated expression of B7-H4 was associated with shorter survival in the cohort (Fig. 6c**,** log-rank *P* = 0.05). In addition, increased expression of the pan T-cell marker CD3- but not of CD8 or CD20 was significantly associated with longer overall survival (log-rank *P* = 0.03, Fig. 6d-f).

**Table 1:**
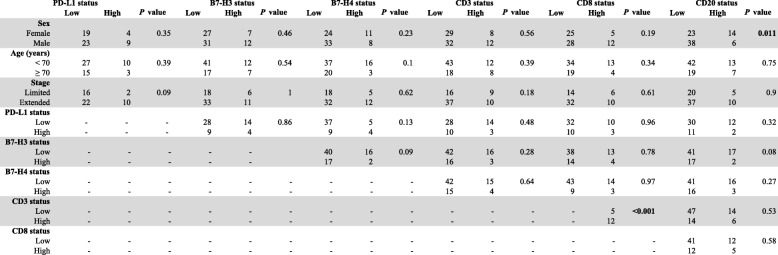
Association of PD-L1, B7-H3, B7-H4 and TIL subsets with major clinico-pathological characteristics and TILs in SCLC

## Discussion and conclusions

Using multiplexed tissue analysis, we have objectively measured 3 different B7-family member ligands and TIL subsets in a sizable collection of human SCLCs. We found relatively low levels of PD-L1, B7-H4 and TILs; but prominent expression of B7-H3 protein. In addition, we found a previously unrecognized negative prognostic role of B7-H4 and a positive prognostic effect of CD3+ TILs in this malignancy. Taken together, our data support that SCLC is a relatively “immune-cold” tumor and suggests the presence of prominent immune regulatory mechanisms. Elevated expression of B7-H3 could mediate immune evasion in SCLC and represent a therapeutic opportunity.

Diverse studies have interrogated the expression of PD-L1 by chromogenic immunohistochemistry (IHC) in SCLC and have reported highly variable results ranging 0–71.6% [14, 15, 24]. These differences could be explained using different IHC assays, analysis platforms and stratification cut-points. One study showing 71.6% PD-L1 expression used a commercial rabbit monoclonal antibody (Abcam, Cambridge, UK) with 5% positive tumor cell as cut-point, but did not specify the clone name and validation status [15]. Two other studies using the validated antibody clone E1L3N [25] and semi-quantitative scoring reported expression frequencies of 0% in tumor cells (0/94 cases) with 18.5% in stromal/immune cells (17/92 cases) [14]; and 5.8% overall PD-L1 expression (4 of 69 cases) [24]. The latter results are similar to our study showing infrequent expression of PD-L1 in SCLC.

Although anti-tumor activity of PD-1 blocking agents has been shown in recurrent SCLC [12, 13], the predictive value of tumor PD-L1 expression in this malignancy is unknown. Future studies directly comparing the clinical benefit of patients with PD-L1 positive and negative SCLCs will be required to clarify this.

Another finding was the common/high expression of B7-H3 and relatively low expression of B7-H4 in the cohort. To the best of our knowledge, this is the first report about the expression of these targets in SCLC. Interestingly, both markers showed minimal co-expression and low association with PD-L1 suggesting a non-redundant/exclusive expression pattern. A similar finding was recently communicated by our group in NSCLC [21]. In SCLCs the expression of PD-L1, B7-H3 and B7-H4 was not associated with the level of CD3, CD8 or CD20+ TILs. However, elevated expression of B7-H4 was significantly associated with worse overall survival supporting a role of this marker in SCLC progression.

Targeting B7-H3 is currently being evaluated as anti-cancer immunostimulatory strategy in preclinical models and in early phase clinical trials [26, 27]. Enoblituzumab (MGA271, Macrogenics) is an Fc-optimized monoclonal antibody to selectively target B7-H3 and is currently in phase 1 studies alone or in combination with PD-1/CTLA-4 inhibitors (NCT02475213, NCT01391143 and NCT02381314). Further understanding of the modulation of B7-H3 expression, identification of its cognate receptor(s) and immuno-modulatory role in cancer will be key to support further clinical development of this pathway.

Our data show that SCLCs display relatively low T- and B-cell infiltration despite being traditionally associated with prominent tobacco exposure, high mutational load and production of neuroendocrine antibodies mediating autoimmune paraneoplastic syndromes [3, 4, 28], In addition, SCLCs have a low total/effector T-cell ratio and limited association between TIL levels and survival. This supports a limited adaptive anti-tumor response in most SCLCs and suggests the presence of potent tolerogenic mechanisms in this malignancy. Possible mechanisms involved in immune evasion are currently unknown but may include an altered tumor microvasculature, epigenetic silencing of immunogenic tumor epitopes, metabolic competition between tumor and immune cells and expression of multiple potent immune suppressive targets/pathways [28]. Additional studies will be required to explore these possibilities. Notably and different to other tumor types [29, 30], only CD3+ but not CD8+ TILs were prognostic in SCLCs. A lack of prognostic value of CD8+ TILs as measured by chromogenic IHC and semi-quantitative scoring was also recently reported in a retrospective cohort of 66 stage I-III lung SCLCs [31]. The limited prognostic value of cytotoxic CD8+ TILs in SCLC could be at least partially explained by the relatively low levels of this immune cell subset. The positive prognostic effect of CD3 could be due to a higher dynamic range of this marker and to the contribution to this score of additional non-cytotoxic CD3+ immune cell populations such as CD4+ TILs and NKT cells.

Our study has limitations. The evaluation of cases was performed using TMAs with possible over/under-representation of the markers due to evaluation of relatively small tumor areas. In addition, tumor tissue was obtained from a single tumor location, limiting the representation of additional lesions not sampled during the diagnostic workup. However, diverse reports measuring immune markers using TMAs from individual tumor lesions have shown consistent results and significant association with clinicopathologic features and outcome supporting the value of this approach [16, 18, 20, 22, 25]. Finally, the cut-points used for marker stratification were based on the relative abundance of the protein signal within the cohort and should be considered as exploratory. Additional studies using independent SCLC collections will be required to validate optimal marker stratification strategies in this disease.

In summary, we have quantitatively measured the expression of 3 different B7-family ligands and major TIL populations in human SCLC. Our data indicate variable expression of the markers with predominance of the candidate immunostimulatory target B7-H3; and the presence of a limited cytotoxic anti-tumor immune response in this malignancy.

## Abbreviations

DAPI: 4′,6-diamidino-2-phenylindole
FFPE: Formalin-fixed, paraffin-embedded
IHC: Immunohistochemistry
LADC: Lung adenocarcinoma
LSCC: Lung squamous cell carcinoma
NSCLC: Non-small cell lung cancer
QIF: Quantitative immunofluorescence
SCLC: Small cell lung cancer
TILs: Tumor-infiltrating lymphocytes
TMA: Tissue microarray
